# Oxidative Stress in the Pathogenesis of Colorectal Cancer: Cause or Consequence?

**DOI:** 10.1155/2013/725710

**Published:** 2013-05-14

**Authors:** Martina Perše

**Affiliations:** Institute of Pathology, Medical Experimental Centre, Faculty of Medicine, University of Ljubljana, Zaloška 4, 1105 Ljubljana, Slovenia

## Abstract

There is a growing support for the concept that reactive oxygen species, which are known to be implicated in a range of diseases, may be important progenitors in carcinogenesis, including colorectal cancer (CRC). CRC is one of the most common cancers worldwide, with the highest incidence rates in western countries. Sporadic human CRC may be attributable to various environmental and lifestyle factors, such as dietary habits, obesity, and physical inactivity. In the last decades, association between oxidative stress and CRC has been intensively studied. Recently, numerous genetic and lifestyle factors that can affect an individual's ability to respond to oxidative stress have been identified. The aim of this paper is to review evidence linking oxidative stress to CRC and to provide essential background information for accurate interpretation of future research on oxidative stress and CRC risk. Brief introduction of different endogenous and exogenous factors that may influence oxidative status and modulate the ability of gut epithelial cells to cope with damaging metabolic challenges is also provided.

## 1. Introduction

There is growing support for the concept that reactive oxygen species (ROS), which are known to be implicated in a range of diseases, may be important progenitors in carcinogenesis [1]. In the last decade, growing number of reports investigating association between ROS and carcinogenesis have been published. Reports have proposed various consequences of oxidative stress that may be linked to carcinogenesis [2–4].

The aim of this review is to briefly summarize proposed mechanisms of oxidative stress that are implicated in carcinogenesis, to review evidence linking oxidative stress with colorectal cancer (CRC), and to provide essential background information for accurate interpretation of future research on oxidative stress and CRC risk. For this reason, we provide brief introduction of different endogenous and exogenous factors that may influence oxidative status and modulate the ability of gut epithelial cells to cope with damaging metabolic challenges.

## 2. Proposed Mechanisms of Oxidative Stress in the Pathogenesis of Cancer

When free radicals are produced in excessive and uncontrollable amounts, they and their derivative products may react with various cellular macromolecules, such as lipids, proteins, and DNA and may modulate gene expression. 

### 2.1. Lipid Oxidation

ROS have the ability to oxidize polyunsaturated fatty acids (PUFAs), which take part in cell membrane constitution. This reaction initiates lipid peroxidation, a chain reaction that produces other free radicals and substances such as malondialdehyde (MDA), conjugated dienes, hydroperoxides, lipoperoxides, and toxic aldehydes [2, 5]. Lipid peroxidation changes the fluidity of cell membranes, reduces the capacity to maintain an equilibrated gradient of concentration, and increases membrane permeability and inflammation [6]. Namely, leakage of normal intracellular enzymes into extracellular fluids results in chemotaxis of neutrophils and other inflammatory cells to the site of injury [7]. In addition, products of lipid peroxidation (particularly MDA and 4-hydroxy-2-nonenal (HNE)) may act as signaling transducers and at low levels modulate several cell functions including gene expression and cell proliferation [2, 8–10]. They also have high reactivity with DNA bases. MDA, which is one of the best known breakdown products of lipid peroxides, was found to react with DNA dG, dA, and dC forming M_1_G, M_1_A, and M_1_C DNA adducts, respectively. These etheno-DNA adducts are mutagenic, and evidence indicates that they may contribute to cancer, including CRC [11–13]. 

### 2.2. Protein Oxidation

Proteins are also susceptible to ROS and are frequent target of increased production of free radicals. ROS oxidize structural proteins and inhibit proteolytic system. Such reactions lead to alteration of structure of proteins or alteration of enzyme functions. The latter can have a wide range of downstream functional consequences, such as inhibition of enzymatic and binding activities, increased or decreased uptake by cells, inactivation of DNA repair enzymes, and loss of fidelity of damaged DNA polymerases in replicating DNA [14]. Oxidized proteins are catabolised in order to reform amino acids. Moderately oxidized soluble cell proteins are selectively and rapidly degraded by the proteasome, while severely oxidized proteins (carbonyl by-products) are poor substrates for degradation and accumulate in cells [15]. It is assumed that accumulation of such damaged material over time contributes to various age-related pathologies in man [14, 16]. Namely, accumulation of damaged proteins in cell acts as an inhibitor of the proteasome, which decreases the capacity for removal of oxidized proteins, accelerates the accumulation of misfolded and damaged proteins, and affects cellular lysosomal system. This in turn hampers protein turnover and gradually leads to further structural and functional alterations of cell organelles [17].

### 2.3. DNA Oxidation

ROS are also known to cause oxidative nucleobase modifications in DNA (i.e., oxidized thymines, oxidized cytosines, oxidized adenines, oxidized guanines), which may lead to carcinogenesis via mispair/mutagenic potential of the modified base. For example, formation of 8-oxodG in DNA leads to G → T transversions during replication unless the damage is repaired by base excision repair (reviewed in [18]). Recent evidence demonstrates that 8-oxodG in the nucleotide pool can be metabolized to form 8-oxodGTP, which can then incorporates into DNA during cellular replication or during DNA repair leading to A → C transversions [19]. The dGTP nucleotide pool is mainly located in the cytoplasm and is thus more available for attack by ROS in comparison to dG incorporated in DNA, which is located in the nucleous and protected by histones [19]. Oxidation of DNA may affect DNA methylation due to oxidation of DNA at either the methylated cytosines or guanines in CpG sequences. DNA adduct formation at the guanine of CpG sequences inhibits binding of the DNA methyltransferase to the adjacent cytosine residue and thus results in hypomethylation of DNA. DNA methylation plays an important role in gene regulation (overexpression or silencing) [18]. DNA hypomethylation occurs in many cancers, including colorectal cancer. 

### 2.4. Modulation of Cellular Signaling

Redox environment is critical factor in cellular signaling. ROS play important roles as intracellular signaling molecules. They are involved in various physiological cellular processes. Under homeostatic conditions, ROS are critical to multiple signal transduction pathways by acting as second messengers. ROS regulate key cellular functions such as proliferation, differentiation, growth, and apoptosis through cellular signaling. Among the most known pathways are NF-*κ*B, the phosphatidyl inositol-3 kinase (PI3K)/Akt pathway, heat shock proteins, and the mitogen-activated protein kinase (MAPK) pathway. However, beneficial or harmful role of ROS depends on their concentrations. Under conditions of oxidative stress where levels of ROS are imbalanced with antioxidants, ROS can be detrimental for the cell itself, leading to uncontrolled proliferation, inflammation, or apoptosis [1, 20–24].

## 3. Oxidative Stress and Colorectal Cancer

Colorectal cancer (CRC) is one of the most common cancers worldwide, with the highest incidence rates in western countries [25]. It is estimated that most of the cases of CRC occur sporadically (70–80%), while approximately 15% of CRC cases develop as a result of inherited factors, such as familial adenomatous polyposis (FAP) and hereditary nonpolyposis colorectal carcinoma (HNPCC) [26]. Changes in worldwide variations in the incidence rates, together with the results of migrant studies, show that sporadic human CRC may be attributable to various environmental and lifestyle factors, such as dietary habits, obesity, and physical inactivity [27]. 

Colon cancer originates from the epithelial cells that line the bowel. These cells divide rapidly and have a high metabolic rate, which has been found as a potential factor that may be responsible for increased oxidation of DNA [28]. Study on primary rat colonocytes has shown that cells from lower crypt sections are more sensitive towards hydrogen peroxide damage than differentiated cells at the surface of the crypt [29]. Since proliferating cells (stem cells and their dividing daughter cells) in the colon are based in the lower part of the crypt, this may show that proliferating cells are putative target cells of colon carcinogenesis. Stem or progenitor cells have been shown to be very sensitive to the redox environment. Their self-renewal and differentiation depend largely on redox environment in the gut mucosa. Proliferating cells are also exceptionally sensitive against DNA damage because the DNA is present as single strand in the S-phase of the cell cycle and serves as template for the complement strand in daughter cells. DNA damage in single strand could lead to varying mutations in the DNA of daughter cells, which could not be repaired [29]. DNA damage can result in either cell cycle arrest or induction of transcription, induction of signal transduction pathways, replication errors, and genomic instability, all of which are associated with colon carcinogenesis [4]. However, recent evidence has suggested that the generation of ROS may play important role in all phases of carcinogenesis, that is, the initiation, promotion, and progression stages [30].

### 3.1. Markers of Oxidative Stress in CRC

In the last decade association between oxidative stress and CRC has been intensively studied. Excellent review of some more commonly used biomarkers of oxidative stress (such as MDA, HNE, acrolein, isoprostanes, glutathione status, tyrosine oxidation, nitration) in human disease and discussion about shortcomings related to validation criteria and other confounding factors has been published by Dalle-Donne et al. [31]. 

It was found that the human colorectal tumors (adenomas and carcinomas) have increased levels of different markers of oxidative stress, such as increased levels of ROS (measured by chemiluminescence), nitric oxide (NO) [32], 8-oxodG in DNA [33], lipid peroxides, glutathione peroxidase (GPx), catalase (CAT) [34], and decreased methylation of cytosine in DNA [33]. Besides lipid modifications also increased leukocyte activation in carcinogenic tissue was found [34], which indicates possible contribution of inflammatory cells to a further oxidative stress [32]. It was found that the level of DNA lesions varied between colon and rectum tissues, being lower in the former than in the latter [35]. 

In addition to colon tumors, significantly increased levels of 8-oxodG in DNA in leukocytes [33] and serum [36] of cancer patients were also detected. Guz and coworkers [33] found significantly decreased methylation of cytosine residues in DNA of leukocytes of patients with colorectal tumors in comparison to the levels found in leukocytes of healthy subjects. 

It was found that whole-blood levels of ROS (measured by chemiluminescence) were higher in patients with history of sporadic CRC in comparison with age- and gender-matched healthy controls. No difference in the whole-blood levels of ROS was found between patients gene carriers for HNPCC or patients with FAP and their corresponding healthy controls. These results suggest that ROS may play a role in the etiology of sporadic cancer. However, since they lack important information about patient's lifestyle habits (smoking, drinking, use of antioxidants, exercise, etc.), additional investigations are needed before any conclusions can be made [37].

## 4. The Protective Mechanisms against Oxidative Stress

**Figure 1:**
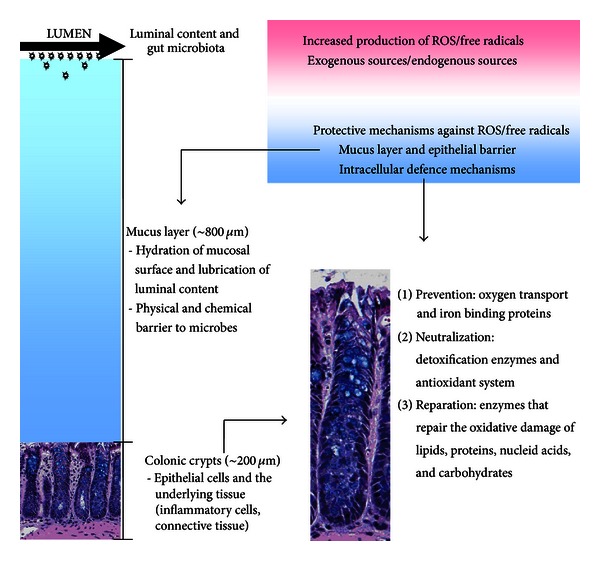
Shematic representation of the colonic barrier and intracellular protective mechanisms against oxidative stress.

All these previously mentioned findings support the hypothesis that oxidative stress may be implicated in colorectal carcinogenesis. However, living organisms are constantly exposed to numerous challenges (in the outer or inner environment) that can significantly affect redox potential of their cells. Therefore, they have developed various protective mechanisms that provide cells with enormous capacity of redox homeostasis. These antioxidative protective mechanisms can be divided into three levels of defence (Figure 1) [38]. 

*The first level* of antioxidative defence is represented by the organization of oxygen transport or by the proteins, which bind iron and in this way prevent the Fenton's reaction. *The second level* includes two primary defense systems, that is, detoxification enzymes that may be controlled by the level of the xenobiotics and antioxidant system that reduce free radical species and maintain the redox state of the cell. *The third level* of defence includes enzymes that repair the oxidative damage of lipids, proteins, carbohydrates and nucleid acids [38]. Some of these enzymes are different proteolytic enzymes, glycosylases, endo- and exonucleases, DNA ligases, DNA polymerases, and so forth. For example, repair and removal of DNA containing oxidized bases in vivo are regulated by DNA glycosylases [19], mainly through the base excision repair (BER) although certain types of oxidative lesions also appear to be repaired by nucleotide excision repair (NER) and mismatch repair (MMR) [18]. Cells that possess complex DNA repair system are composed of BER, NER, global genome repair (GGR), and the transcription-coupled repair (TCR) [39]. Failure in these protective mechanisms may represent one of the risk factors in the etiology of CRC.

### 4.1. The Role of Protective Mechanisms in Oxidative Stress Induced CRC

The antioxidant defence system is known to be composed of numerous antioxidants which work collectively. Antioxidants are divided into primary (superoxide dismutase (SOD), CAT, GPx, glutathione reductase (GR)), secondary (vitamin E, vitamin C, beta-carotene, uric acid, bilirubin, and albumin) and tertiary (biomolecules damaged by free radicals) defence elements in the cell [40]. Thus, micronutrient antioxidants may have by virtue of their free radical scavenging properties important role in the redox homeostasis. Patients with adenomatous polyps had significantly lower levels of all measured micronutrient antioxidants (*α*- and *γ*-tocopherol, lutein, *β*-cryptoxanthin, lycopene, and *α*- and *β*-carotene) in their colon mucosa than their healthy control subjects. However, their serum levels of these antioxidants were similar in both groups [41]. In contrast, another study reported that colorectal cancer patients had significantly decreased levels of antioxidant enzymes, vitamins C and E in the serum than corresponding healthy control group [36]. Low intake of the micronutrient selenium (Se) has been implicated as a risk factor in CRC. Epidemiological studies linking Se intake to CRC risk have found strong evidence for a link to adenoma risk [42]. 

Much of research has recently been focused on the investigation of genetic factors that may affect susceptibility to CRC. Several single-nucleotide polymorphisms (SNPs) in genes implicated in antioxidative protective system, such as eosinophil peroxidase, myeloperoxidase [43], SOD2 (MnSOD), and selenoprotein, have been found [42, 44]. Selenoproteins are group of ~25 proteins with incorporated selenocysteine. They are implicated in various protective mechanisms against oxidative stress. For example, the GPx are antioxidative enzymes, thioredoxin reductases (TR) function in redox control, selenoprotein P (SePP) transports selenium to tissues, and selenoprotein S (SelS) is involved in removing unfolded protein response [42]. 

It was found that genetic variation in various selenoprotein genes may influence susceptibility to CRC. For example, study on 832 CRC patients and 705 healthy controls showed significant association between SNPs in *SEPP1*, *GPX4*, and *SELS* genes and risk of CRC [42]. Another study on 827 patients with CRC and 733 healthy controls found association between SNPs in *SEP15* and *SELS* genes and altered risk of CRC [44]. These SNPs have been shown to have functional consequences. It was suggested that these variants play a role in cancer development and may thus represent potential biomarkers for CRC risk. Furthermore, findings from two population-based case-control studies of colon (*n* = 1555 cases, 1956 controls) and rectal (*n* = 754 cases, 959 controls) cancer support an association between selenoprotein genes and CRC development and even survival after diagnosis. Results also suggested that the impact of cancer susceptibility from genotype may be modified by lifestyle [45]. Importance of antioxidative protective system has been recently demonstrated on animal model. Induction of inflammatory colon carcinogenesis in GPx-3 deficient mice resulted in an increased tumor number along with a higher degree of dysplasia, increased inflammation, increased proliferation, hyperactive Wnt signaling, and increased DNA damage [46]. In addition, genetic variation in the MAPK signaling pathway, downstream target for ROS, has been shown to be associated with CRC risk and survival after diagnosis [47]. There are also studies investigating diet-gene interactions and the mechanisms by which food components regulate gene expression to modify CRC susceptibility [48].

## 5. Sources of Free Radicals in Colon: Beneficial, Harmful, or Confounding

Human colonic contents are diverse mixture of bile, mucus, desquamated epithelial cells, various microorganisms and their fermentation products, undigested or unabsorbed food and its metabolic products, such as metals, salts, toxins, mutagens, carcinogens, and dissolved gases (like nitrogen, hydrogen, carbon dioxide, methane, oxygen). It is believed that intestinal mucosa is constantly challenged with diet- and bacterial-derived oxidants and carcinogens. Chronic exposure of such challenging conditions may then lead to uncontrolled generation of free radicals, redox imbalance, and DNA damage, which can affect intestinal metabolic homeostasis with cancer as an endpoint [33].

It is noteworthy to recognize that epithelial cells in the gut mucosa of a healthy individual are not in direct contact with the luminal content or gut microbiota. The secreted mucus layer is very thick (~800 *μ*m in the rodent colon) and represents both a physical and chemical barrier to microbes. Its function is also to keep the mucosal surface well hydrated and to lubricate luminal content [49]. Gut mucosa is composed of a thick secreted mucus layer, a layer of epithelial cells and the underlying nonepithelial tissue, composed of inflammatory cells, connective tissue, and so forth (Figure 1) [49]. All these components are intrinsically linked in a complex physiology. 

In addition, the gut mucosa is not constantly exposed to harmful challenges. The gut mucosa is exposed to various beneficial and modifying factors (e.g., healthy food, exercise) that can counteract deleterious effects of harmful challenges. To get an insight into this comprehensive and complex field, brief introduction of harmful, beneficial, and modifying effects of some endogenous and exogenous factors on antioxidant status are represented in the following section.

### 5.1. Gut Microbiota

The gut microbiota (termed microflora) contains a broad spectrum of microorganisms, which resides in the gastrointestinal tract and play important role in human health and disease (reviewed in [50]). They are essential for the host's wellbeing in terms of nutrition and mucosal immunity. Certain members of the gut microbiota have been shown to promote the host's health. However, there are also numerous studies that have implicated some members of the gut microbiota in the development of CRC due to different mechanisms including generation of reactive metabolites (*E. faecalis* produces hydroxyl radical-potent source of oxidative stress on the intestinal epithelium) [51] and carcinogens, alterations in host carbohydrate expression and induction of chronic mucosal inflammation [52]. The human intestinal habitat contains 300–500 different species of bacteria, varying significantly in content between individuals, which may potentially represent huge variability in the formation of free radicals among men.

### 5.2. Inflammation

Rapidly growing body of evidence indicates that chronic inflammation is important factor in development of carcinogenesis [7, 53]. It is widely known that CRC is a complication of a chronic inflammatory state in the bowel. Patients with inflammatory bowel disease (ulcerative colitis or Crohn's disease) have 6-fold increased risk to develop CRC compared with the general population [46]. Excessive and uncontrollable production of ROS for a longer period of time results in persistent injury of cells in the tissue and consequently persistent inflammation. Besides damaged cells also inflammatory cells produce soluble mediators, which act by further recruiting inflammatory cells to the site of injury and producing more reactive species [53, 54]. This sustained inflammatory/oxidative environment leads to an enhanced production of hydroperoxides in a vicious circle, which can damage healthy epithelial and stromal cells in the vicinity of injury and over a long time may lead to carcinogenesis. The role of chronic inflammation and oxidative stress in carcinogenesis is excellently explained elsewhere [7, 53]. 

### 5.3. Food

It has been demonstrated that dietary fatty acids affect the lipid content of tissue and result in differential susceptibility to peroxidation [55–58]. Lipids and fatty acids obtained from dietary fats are metabolized and incorporated into the phospholipids of the cell membranes of many cell types and serve as precursors for many biologically active molecules, as well as being important for cell signalling or different intensity of inflammation response [59, 60]. A substantial increase in the PUFA content may overcome the protective action of the antioxidant system and increase susceptibility to lipid peroxidation [61]. We have recently demonstrated that long-term consumption of an high-fat mixed-lipid (HFML) diet significantly increased the production of lipid peroxides in the liver [62] and skeletal muscle [63] and increased development of CRC [62]. On the other hand, fish oil has been found to reduce oxidative DNA damage [58, 64]. It was recently shown that a high-fat, low-calcium, and vitamin D diet induces oxidative stress in the colon [65]. Hemoglobin from either red meat or bowell bleeding may act as an enhancer of oxidative damage in the bowel [66].

On the other hand, micronutrient antioxidants may have by virtue of their free radical scavenging properties important role in the redox homeostasis. Nutritionally derived antioxidants such as vitamin E and C, beta-carotene [67], flavonoids and polyphenoles may provide second line of defence against the production of ROS. Epidemiological studies evaluating the occurrence of polyps after supplementation with vitamin E and *β*-carotene have yielded mixed results [41], while experimental studies demonstrated protective effects. It has been already shown that vitamin E has antiproliferative properties in cancer cell lines, while different natural antioxidants such as gallic acid [68], polyphenols [69], vitamin D [70], vitamin A [71] pharmacological compounds like bis-1,7-(2-hydroxyphenyl)-hepta-1,6-diene-3,5-dione (BDMCA) [72] have potential to inhibit CRC development in dimethylhydrazine (DMH) model, which is well-established CRC animal model and possesses many characteristics found in human sporadic CRC [73].

### 5.4. Obesity

Obesity, particularly abdominal obesity, was associated with increased risk of CRC and was found to affect oxidative status in obese people [74–77]. It is known that adipose tissue produces various adipocytokines (e.g., adiponectin, leptin, and numerous cytokines such as TNF*α*, IL-6, IL-8, and IL-10) that are implicated in normal functioning of the body. Evidence has shown that increasing obesity alters levels of adipocytokines, increases circulating oestrogens, decreases insulin sensitivity, and raises the inflammatory response. It causes so-called metabolic syndrome or low-grade chronic inflammation, which may be responsible for constant increase in production of free radicals. It is assumed that over time such conditions (oxidative stress) create environment favorable to the CRC development [74–77].

### 5.5. Aging

Accumulated evidence suggests that aging is associated with increased production of free radicals, resulting in increased oxidation of lipids, proteins, and genetic material [78]. Oxidative conditions cause progressive structural and functional alterations of cellular organelles and changes in redox-sensitive signalling processes [40, 79, 80]. Such cellular conditions contribute to increased susceptibility to a variety of diseases, including inflammation and cancer [81]. Oxidative stress as a consequence of increased production of nitrogen or oxygen reactive species has been demonstrated in inflammatory bowel disease and CRC.

### 5.6. Physical Activity

Regular exercise may help to prevent colon cancer due to an improvement in the cell's antioxidant defence system. It has already been demonstrated that exercise improves the antioxidant defence system in various tissues. Exercise stimulates various signaling pathways in cells, such as MAPK and NF*κ*B, which results in increased expression of important enzymes associated with cell defence (MnSOD and GPx) and adaptation to exercise (eNOS and iNOS) [21, 22]. Many of the biological effects of antioxidants appear to be related to their ability not only to scavenge deleterious free radicals but also to modulate cell-signalling pathways. The modulation of signalling pathways by antioxidants could thus help prevent cancer by preserving normal cell cycle regulation, inhibiting proliferation, inducing apoptosis, inhibiting tumor invasion and angiogenesis, suppressing inflammation, and stimulating detoxification enzyme activity [30, 82, 83]. Exercise has been found to decrease the expression of inducible nitric oxide synthase (iNOS), as well as TNF-*α*, in the colon of azoxymethane- (AOM-) treated mice [84].

## 6. Concluding Remarks

As demonstrated previously, gut mucosa possesses various protective mechanisms to neutralize effects of increased production of free radicals, that is, thick secreted mucosal layer, which represents important physical and chemical protective defence to luminal content and strong antioxidative protective mechanism. We provided data demonstrating that oxidative status and ability of antioxidant system to respond to various conditions are influenced heavily by a number of physiological and environmental factors. Various redox-dependent mechanisms in an organism could be affected by different endogenous or exogenous factors with protective (scavenging), harmful (accelerating), or modifying effects on production of free radicals. Recent studies have found an association between a genetic variant in some genes of antioxidative protective mechanisms and CRC risk. On the basis of the known and suggested role of previously mentioned proteins (selenoproteins, SOD, etc.) in cell protection mechanism, it is possible that factors that affect their pattern of colonic expression may modulate the ability of gut epithelial cells to cope with damaging metabolic challenges. For instance, low dietary Se intake or genetic variation in the selenoprotein genes may influence expression or function of selenoproteins, respectively. As demonstrated, these studies represent ground for various speculations. Thus, before we can answer the question whether oxidative stress is a cause or a consequence of cancer development, further studies elucidating the role of antioxidative protective defence and other confounding factors in the pathogenesis of CRC are needed.
